# Beyond validation: getting health apps into clinical practice

**DOI:** 10.1038/s41746-019-0212-z

**Published:** 2020-02-03

**Authors:** William J. Gordon, Adam Landman, Haipeng Zhang, David W. Bates

**Affiliations:** 1 Division of General Internal Medicine and Primary Care, Brigham and Women’s Hospital, Boston, MA USA; 2 Partners HealthCare, Somerville, MA USA; 3 Harvard Medical School, Boston, MA USA; 4 Department of Emergency Medicine, Brigham and Women’s Hospital, Boston, MA USA; 5 Department of Psychosocial Oncology and Palliative Care, Dana-Farber Cancer Institute, Boston, MA USA; 6 Brigham and Women’s Hospital, Boston, MA USA

**Keywords:** Signs and symptoms, Diagnostic markers, Health services

## Abstract

Fueled by advances in technology, increased access to smartphones, and capital investment, the number of available health “apps” has exploded in recent years. Patients use their smartphones for many things, but not as much as they might for health, especially for managing their chronic conditions. Moreover, while significant work is ongoing to develop, validate, and evaluate these apps, it is less clear how to effectively disseminate apps into routine clinical practice. We propose a framework for prescribing apps and outline the key issues that need to be addressed to enable app dissemination in clinical care. This includes: education and awareness, creating digital formularies, workflow and EHR integration, payment models, and patient/provider support. As work in digital health continues to expand, integrating health apps into clinical care delivery will be critical if digital health is to achieve its potential.

## Background

Digital technology offers tremendous potential for improving the prevention, diagnosis and management of disease. The proliferation of health apps in particular—there are now more than 300,000 which have been developed^1^—is changing how patients interact with the healthcare system. Through apps, patients can have immediate access to their health data, schedule virtual visits with their providers, integrate with devices like blood pressure cuffs, manage medication dosing, improve wellness, and many other health-related activities. Apps are also increasingly accessible especially via smartphones—more Americans now have a smartphone than a desktop or laptop computer.^2^

Health apps exist on a spectrum: from consumer facing, non-regulated, non-interventional apps like fitness trackers, to regulated, prescription-only apps like a digital therapeutic to manage substance use disorder. Some are standalone apps, while others require connection to an external device, like an inhaler. Here, we consider apps that are used by individuals, typically on a mobile device such as a smartphone or smartwatch, to support many aspects of health—including, diagnosis, treatment, and monitoring. Similar to the US Food and Drug Administration (FDA) definition of a “Mobile Application,” we define “apps” as software applications that may run on a variety of mobile platforms or be web-based but optimized for mobile devices.^3^ There is significant work underway to develop apps, build an evidence base, validate functionality, create standards for development, and design frameworks for app evaluations.^4–8^ Capital is pouring in—more than $9 billion was invested by venture capital and private equity towards digital health companies in 2018, $2 billion more than 2017.^9^ Additionally, the FDA recently launched a Digital Health Innovation Action Plan.^10^ A key component of this plan is a new regulatory pathway for certain software devices called “precertification” that is intended to streamline approval by focusing on application developers and processes as opposed to specific products.^11,12^ In 2018, the World Health Organization published guidelines on digital health interventions,^7^ and recently, the UK National Institute for Health and Care Excellence published an evidence framework for digital health technologies.^13^

However, while advancements have been made in the development, validation, and regulation of apps, it is less clear how to disseminate appropriate interventions to patients and providers.^14^ The sheer number of apps available, each with varying functionality, complexity, impact, and cost, creates substantial barriers to the diffusion of these apps into clinical care. Furthermore, the evidence base supporting the use of even the best apps is scant, and most apps do not deliver value, especially for patients who are sick or have chronic conditions.^15^ Some apps may even be harmful.^16^ Yet even if high-quality apps are developed, the potential of apps to improve the care and wellbeing of patients can be realized only if the tools are actually used. If they are to be effective, both patients and providers need to gain value from utilizing these tools. It is also critical that they connect with other digital applications such as the electronic health record, yet most do not today.

One potential solution is to frame apps like non-digital treatment modalities, such as medications. If apps could be “prescribed” to patients through existing workflows, patients and clinicians may be more likely to use them, and patients could be steered through the maze of apps today towards ones which are most likely to be beneficial. Such a model promises better integration of apps into clinical practice, but also raises new issues around awareness, process, technical support, and payment.

In this review, we first describe the current regulatory environment in both the US and Europe around apps and mobile technology. We then discuss the importance of validation, and how to ensure apps that are used in clinical practice have been appropriately validated. We then build a framework for prescribing apps and outline the key issues that need to be addressed—along with potential solutions—to truly enable apps to become a core component of clinical care.

## Regulatory environment

Both the US FDA and the EU, largely through the European Medicine Agency (EMA), have long recognized the importance of software’s role in diagnostic and therapeutic devices.^17,18^ More recently, to address the increasing importance of digital health, the US FDA launched a Digital Health Innovation Action Plan.^10^ Run through the Center for Devices and Radiological Health, the plan includes several areas of updated or new guidance, additional personnel and digital health expertise at the FDA, and a “precertification” program to streamline device approvals by focusing on developers and processes as opposed to specific products. The FDA considers two main subsets of device software functions: Software as a Medical Device (SaMD) and Software in a Medical Device (SiMD). Software that ultimately meets the definition of a device can be deployed on a mobile platform, at which point it is defined as a “mobile medical app.”^3^ As such, in the US, regulation of mobile apps follows guidelines set by the Federal Food, Drug, and Cosmetic Act (FD&C Act), recently updated by the 21st Century Cures Act to exclude certain software capabilities. In September 2019, the FDA updated its guidance on Device Software Functions and Mobile Medical Applications to further clarify its oversight role for software.^3^ Importantly, the guidance documentation indicates that the FDA will not enforce requirements for software (and thus medical apps) that (1) help patients self-manage their disease without suggesting specific treatments, and (2) automate simple tasks for healthcare providers. In the US, additional examples of federal oversight include the Federal Trade Commission’s Health Breach Notification Rule and the Health Insurance Portability and Accountability Act.^19^

Similarly, in Europe, there have been multiple efforts to provide frameworks and regulatory best practices in this space. The European Commission eHealth Action Plan 2012–2020, for example, set out a policy roadmap and “digital agenda” for eHealth in Europe.^20^ Regulation (EU) 2017/745 on Medical Devices (MDR), which began in 2017 and will fully apply in May 2020, is the most recent EU framework for medical devices. As in the US, “apps” are vehicles for software deployment, and thus whether software qualifies as a device is independent of where it is deployed.^21^ The EU General Data Protection Regulation (GDPR), which went into effect in May of 2018, also provides important guidance for app developers, particularly around data protection and privacy.

## Clinical value and validation

Traditional regulatory models have an important role in making certain apps available for clinical care, and we anticipate that these regulated apps will also have evidence supporting their use, such as a randomized control trial. However, as noted above, the majority of apps will not be tightly regulated. For these apps, different models of validation will be needed so that clinicians and patients can understand which apps deliver value and which do not—i.e. some measure of quality and safety, like clinical benefit, patient satisfaction, potential adverse effects, provider burnout mitigation, or cost-effectiveness. While some apps have been rigorously studied, there is a general dearth of evidence for health apps, both because a small percentage have been studied, and evidence tends to be low quality for those that have been studied.^8,22–24^

Numerous strategies have been proposed to improve the validation and trustworthiness of apps, such as having a voluntary accreditation agency that will “certify” apps, provider-based efforts to validate apps, or independent third-party reviewers, among others.^8,25,26^ While consumer ratings of health apps might seem to be a scalable methodology of validating apps, prior work suggests that consumer ratings poorly reflect clinical utility and usability.^15^ Given the scale of non-regulated app development, we anticipate that multiple strategies will be needed, and many stakeholders will be involved. Clinicians will play an integral role, particularly given the trust that many patients place in their providers. Additionally, many apps will be marketed directly to patients. For these apps, industry and existing regulations (like the Federal Trade Commission in the US) will be essential for ensuring a base level of quality and minimizing erroneous claims. Researchers will play multiple roles—from providing the primary data to support the clinical utility of an app, to uncovering unvalidated and dangerous apps. Though many groups will be involved, the medical community needs to work with the regulatory authorities to help define best practices and identify robust approaches for app validation.

## Education and awareness

Despite the challenges in developing and validating apps, we anticipate that over time more and more apps will clear these hurdles and become appropriate for clinicians to bring into clinical practice. A first step in disseminating apps for clinical use is increasing education and awareness of the available technologies for clinicians. While pharmacology is a core component of medical education, few clinicians receive formal digital health training^27^, though there are efforts to improve this through new initiatives like the American Medical Association’s “Accelerating Change in Medical Education Consortium.”^28^ Other educational approaches are needed to reach practicing clinicians, such as formally requiring digital health training as part of ongoing professional education or certification programs, like Continuing Medical Education, Maintenance of Certification, or board certifications.

Education is particularly salient for prescription-only FDA-regulated apps, which may have specific indications or pre-requisites for appropriate use, like an online cognitive behavioral therapy tool to support outpatient substance use disorder programs.^29^ There are several potential ways to implement this required training. First, special licensing could be required for prescribers, similar to a practice waiver for buprenorphine therapies. While this would create a substantial barrier to more widespread app prescriptions, it would create an ecosystem of mandatory certification and training, which might be beneficial if an app is particularly complex. Lighter-touch approaches, like vendor or hospital-led educational courses, would be another method of disseminating training. App vendors could maintain registries of “certified” providers, which would have the added benefit of alerting interested patients to clinicians that are familiar with a particular app.

Patients can also benefit from education and awareness. As apps become more prevalent, we expect more direct-to-consumer marketing of apps, which is not surprising given the vast majority of apps are designed for consumer usage.^30^ App “stores” represent the primary repository of health apps, and patients rely on how these stores sort and present apps for what is most relevant. However, prior work has also shown that consumer ratings do not correlate with clinical utility.^15^ One proposed solution involves using an “app label” (similar to a nutrition label for food) to help consumers understand the technology and data used by the app.^1^ Regardless of the strategy, ensuring appropriate messaging will be critical, which may require governmental regulatory action, similar to efforts around traditional medication marketing.^31^

## Digital formularies

Traditional formularies are lists of drug products that enable a provider organization, pharmacy, or payer to distinguish between preferred or non-preferred drugs based on several factors including cost and clinical value.^32^ In some settings, formularies dictate what medications are available to be prescribed; in other settings, formularies are used to get lower prices—for example, preference for a particular proton-pump inhibitor based on negotiation. Given the sheer number of available apps, a formulary for health apps—a “digital formulary”—could be an important mechanism for enabling apps to disseminate into clinical care, and would be valuable for the entire spectrum of apps—monitoring, diagnosis, therapeutics, etc. Express Scripts, a large US-based pharmacy benefits manager (PBM), recently announced a digital health formulary.^33^ CVS Health also recently announced a product that would enable its PBM customers to manage third-party health products.^34^ Outside the US, the United Kingdom’s National Health Service has launched an “Apps Library,” which is a curated list of health apps, with pricing information, for patients and providers to search for apps which may benefit them.

Digital formularies serve multiple purposes. First, they provide a short list of available apps, and providers could search these formularies and know what was available for a specific diagnosis or purpose—far more manageable than sifting through 300,000-plus apps in an app store. Second, from a safety perspective, a digital formulary could utilize a higher bar for listing an app than the app store, and could for example include apps which have received regulatory approval, or apps with clear evidence supporting their use. Third, digital formularies could enable streamlined coverage and pricing workflow, like traditional medication formularies. Fourth, digital formularies could provide a mechanism for patients and providers to know which apps would be supported by the provider organization and might for instance interoperate with the EHR they use. Organizations could even offer online, telephone, or in-person support for listed apps.

Digital formularies have two main risks. First, while formularies should overall result in lower pricing for patients, there could also be situations where prices go up, for example, through exclusivity deals, or simply because the digital formulary contains an older, more expensive app, and a newer, cheaper one is available. Second, while narrowing the set of available apps will be helpful, digital formularies could also slow diffusion of apps into practice. If providers and patients rely on these formularies as their primary repository, newer, more effective apps will need to overcome the hurdle of getting into a digital formulary to be used. Thus, one of the main advantages of apps—the speed at which they can be developed and propagated—could be limited by digital formularies. However, this would likely also incent app developers to perform trials of their technologies which could make it easier to identify which are beneficial.

## Workflow and EHR integration

Many providers spend less than 20 min with a patient per outpatient visit, with numerous competing interests for what gets addressed during these visits.^35,36^ Since managing apps will be another competing interest, the process of prescribing apps to patients must be integrated into current provider workflow in order to scale—otherwise, usage will be limited to early champions who dedicate time to learning how to disseminate these apps to their patients, but won’t be accessible to the majority of clinicians who may not prescribe apps because that process exists outside their current workflow.

There are several steps that must be accomplished to integrate apps into provider workflow. First, apps should be searchable (for example, from a digital formulary), and then orderable, from the EHR, just like a medication. Second, apps should be integrated with clinical decision support systems to ensure appropriateness. Third, providers should have the ability to note the indication for the app—why it is being prescribed for that patient—so that other providers understand why the app is being used. This information could also be brought into provider documentation for that patient, either in the medication history or in a new “digital tools” section of a patient note. Fourth, providers should be able to enter the “sig”—or label for the prescription. Fifth, providers should be able to prescribe parameters for the app, which can be loaded automatically once the patient downloads the app. Sixth, the app prescription should be visible in the EHR like other prescriptions, so that other providers know this app has been prescribed and ideally whether or not it is being used by the patient. Seventh, data generated by the app should be accessible to patients and providers, ideally through existing communication channels like patient portals or other interoperability channels. The prescribing provider might need to commit to following any app-related output, for example, through a service-level agreement. Finally, there should be mechanisms to de-prescribe an app, for example if the app is no longer supported, used or indicated. Table 1 highlights each of these steps, with a clinical example.

**Table 1:** Prescribing apps as a component of clinical workflow. A smoking cessation app is used as an example.

Workflow Component	Description	Example
		45 y/o male presents to his PCP interested in smoking cessation
Searchable and Orderable	• Provider searches for an app within their EHR application • The search cross-references the organization’s digital formulary and the patient’s pharmacy benefit digital formulary to ensure access and coverage • Provider selects the app and opens an order screen	• Provider searches for “Smoking Cessation” and finds a set of available, covered apps that address smoking cessation. Provider selects an approved smoking cessation app and enters the order screen.
Clinical Decision Support Integration	• Apps trigger EHR clinical decision support rules • Rules can check for clinical appropriateness, duplicate therapies, other contraindications	• CDS fires and checks that the patient is listed as a current smoker and is not currently prescribed a different app.
App Indications	• Provider enters indication for the app • Indication is visible to other providers in the EHR	• Provider enters “Smoking Cessation” under indication.
App Directions (the digital “sig”)	• Specific directions for app usage are entered, similar to traditional medication “sig” • Apps may have a list of default sigs, similar to how medications often have default common dosing instructions	• Please install application on your smartphone device and use 3 times daily for 6 months.
App Parameterization	• Certain apps may allow for app parameters, which provide settings for the app • Apps will likely have default parameters to select from	• Provider confirms default parameters—for example, “run in background”.
EHR Visibility	• Once ordered, apps are visible in the EHR, so that other providers can see a list of prescribed apps, along with their indication, directions, and parameters • Historical apps can be “re-activated” if clinically appropriate	• Once prescribed, smoking cessation app shows up in the patient’s list of current medications and therapies.
Data Integration	• App results can be surfaced to providers and patients through existing communication channels, like a patient portal or EHR • Data includes app usage (if acceptable from a patient privacy perspective) and any output	• Overall smoking trends and number of cigarettes smoked / prevented are displayed in tabular and graphical formats. • Patients can see this through the app, or through their patient portal. • Provider can monitor patient usage of the smoking cessation app.
De-prescribe	• Apps can be de-prescribed, for example, if they are no longer effective or now contraindicated • Apps can be re-activated in the future	• Patient achieves smoking cessation and app is removed from list of active medications.

The implementation of workflow-based app EHR integration should follow existing paradigms that are in use for traditional medications, as outlined in the smoking cessation app example (Table 1). Exceptions include app-specific concerns that will require more customization, like how to add parameters to a prescription. Enabling bidirectional data flow (so that the app can provide data back to providers and vice versa) would require new processes and new data integrations. At our institution, for example, we work with a vendor that aggregates all incoming device data into a separate patient-specific area of our EHR. Additionally, federally certified EHRs, as a result of national incentive programs and the 21st Century Cures Act, are now required to allow patients to download their data directly through Application Programming Interfaces (APIs), which provides an important, provider-independent mechanism for data exchange.^37–39^

Many apps may be useful for a short period, like preceding a colonoscopy or a surgical procedure. Others may be prescribed for chronic diseases—for the latter both the patient and provider should be able to track how often the patient is engaging with the app and how this relates to control of their illness. Importantly, many apps will be used by patients independent of provider recommendation or prescription (similar to an over-the-counter medication). There should also be mechanisms for providers to indicate in the EHR that a patient is using an app, for what indication, and for how long. Ideally this could be done automatically by a patient. Technologies to enable some of these features are starting to emerge; some examples include vendors such as Xealth and Rx.Health. We anticipate that EHRs will increasingly enable this as core functionality as well.

## Payment models

Apps, like other diagnostics and therapeutics, will need to be paid for. In the simplest model, app fees are paid by patients. However, as apps become more complex, and pass through regulatory hurdles, we expect prices to increase, and patients will seek payment coverage (through public or private payers as appropriate) for these apps. Without reimbursement models, app adoption will struggle as many patients will be unable to pay for them.

Establishing clear reimbursement pathways for apps is critical for several reasons. First, if a pathway for reimbursement is not established, apps could be free, but rely on advertisements or data mining as primary revenue sources. In addition to significant privacy concerns, this will also make apps less useable by patients and create suboptimal incentives for app development. Second, given that apps are usually optimized for smartphones, there is risk of exacerbating a digital divide between patients that have smartphones and those that do not—apps that then are inaccessible due to cost will only deepen that divide. Third, without clear reimbursement models, app makers may be less incentivized to innovate and create new technologies. Finally, apps present unique reimbursement challenges, for example, how updates or new software versions are managed and paid for.

Fortunately, there are also opportunities to innovate. For example, apps could have “trial” periods that would allow a patient to try it out before committing. Payment could also be used to incentivize usage—for example, removing copays if patients demonstrate app usage or achieve app-related outcomes. Accountable care organizations might pay for apps for at-risk patients if the app could reduce total medical expense for those patients. Other apps could follow traditional payment models, like using CPT codes that are reimbursed by insurers.^40–42^ Apps could also be packaged with other medical products, like drugs or devices. Regardless, establishing clear payment and reimbursement models will be essential for apps to become a larger part of medical care.

## Patient and provider support

Finally, as apps become part of clinical workflow, we anticipate an increasing need for user support. Most medications require minimal training—patients are given pills, with instructions on when to take them, with occasional nuance around timing (for example, taking thyroid replacements 60 min before breakfast). Some medications require more education. Inhalers, for example, are commonly used incorrectly, which can lead to worse outcomes.^43^ Pharmacists represent an additional expert resource available to patients with questions about medications. Apps may require even more education. Providers will need to know how to use an app they are prescribing and will need to stay abreast of updates. We expect patients to reach out to clinicians for assistance with prescribed apps, so providers and office staff will need new workflow for managing these types of interactions. Additionally, entirely new professional groups could form with expertise in this area. The recently formed Digital Medicine Society^44^ is one step in this direction.

Similarly, patients will need resources for support. The Ochsner health system created the “O Bar”—a physical space that patients can go to obtain recommended digital interventions as well as troubleshoot digital devices. The O Bar includes mechanisms to test apps and receive digital devices, like Bluetooth-enabled glucose monitors or wireless scales.^45^ Similar efforts have been implemented at MedStar Health in Maryland, Morristown Medical Center in New Jersey, and Sibley Memorial Hospital in Washington D.C.^46^ App vendors also play a role here, and many have their own dedicated support functions. Another possibility is to use the support structure that hospitals have in place for their patient portals. Regardless, building an infrastructure to support installation and usage of apps will be essential as apps become more commonly used.

## Discussion

Digital health, and “apps” in particular, holds tremendous potential for improving health outcomes. But while hundreds of thousands of apps have been developed, most of them to date are rarely used, not clinically validated, and have not been integrated into practice on a broad scale. We outline five key areas that need further development for apps to be integrated into clinical practice and to bridge the divide between the potential of apps and actual clinical use.

We have focused on apps, but there is a broader world of digital health that will face many of the same challenges. Examples include hardware that are dependent on apps, like an app-based portable EKG, or continuous glucose monitors that feed results back to an app. Similarly, the FDA is exploring its role in regulating Prescription Drug-Use Related Software (software that is used in conjunction with a medication, which could be used to send administration reminders, track intake, etc.)^47^ Data aggregators, like Apple’s Health Records on iPhone^48^ enable other apps to utilize EHR data, and could become requirements for specific types of apps to function. In all cases, careful consideration as to how these technologies go from validation to use will be essential.

## Limitations

While apps remain a promising new care delivery tool, there are additional factors that need to be addressed. First, apps usually require smartphones or tablets. Patients who are unable to afford, access, or use these technologies will be unable to benefit from this technology. There is strong evidence that certain populations already experience this digital divide—for example, patients with low health literacy are less likely to use health information technology,^49^ minorities and patients of lower socioeconomic status are less likely to use patient portals,^50^ and users of mobile technology specifically are more likely to be younger, higher educated, male, and reside in zip codes with higher median income,^51,52^ though there is evidence that smartphone ownership is increasing in low-income and low-education populations.^53^ Further work is needed to understand how to close this divide further so that all populations are able to benefit from new digital health tools. Another important consideration is how these apps will impact clinician burnout, which has become an increasing concern amidst evidence of harm to both clinicians and patients.^54,55^ EHRs are often linked to physician burnout,^56,57^ so more digital tooling could exacerbate this concern. However, health apps could also lessen physician burnout, if, for example, apps streamlined communication or made follow up less time intensive. Regardless, more work is needed to understand how apps will affect clinician burnout.

## Conclusion

As work in digital health continues to expand, we expect more apps to become available, some of which will have evidence of efficacy and regulatory approval. Development and validation are just the first steps. For apps to be used, they must be integrated into clinical practice. We have outlined some of the key areas that will need to be addressed: education and awareness, digital formularies, workflow integration, payment models, and patient/provider support. Integrating apps into routine clinical practice will be essential for digital health to achieve its full potential.

## Data Availability

Data available on request from the authors.
